# In-depth characterization of a mouse model of postoperative atrial fibrillation

**DOI:** 10.20517/jca.2022.21

**Published:** 2022-07-19

**Authors:** Joshua A. Keefe, Jose Alberto Navarro-Garcia, Li Ni, Svetlana Reilly, Dobromir Dobrev, Xander H. T. Wehrens

**Affiliations:** 1 Cardiovascular Research Institute, Baylor College of Medicine, Houston, TX 77030, USA; 2 Department of Integrative Physiology, Baylor College of Medicine, Houston, TX 77030, USA; 3 Division of Cardiology, Department of Internal Medicine, Tongji Hospital, Tongji Medical College, Huazhong University of Science and Technology, Wuhan 430030, Hubei, China; 4 Division of Cardiovascular Medicine, Radcliffe Department of Medicine, University of Oxford, John Radcliffe Hospital, Oxford OX3 9DU, UK; 5 Institute of Pharmacology, University Duisburg-Essen, Essen 45122, Germany; 6 Department of Medicine and Research Center, Montreal Heart Institute and Universite de Montreal, Montreal, QC H1T 1C8, Canada; 7 Department of Medicine, Baylor College of Medicine, Houston, TX 77030, USA; 8 Department of Neuroscience, Baylor College of Medicine, Houston, TX 77030, USA; 9 Department of Pediatrics, Baylor College of Medicine, Houston, TX 77030, USA; 10 Center for Space Medicine, Baylor College of Medicine, Houston, TX 77030, USA

**Keywords:** Atrial fibrillation, cardiothoracic surgery, biological sex, inflammation, fibrosis

## Abstract

**Introduction:**

Postoperative atrial fibrillation (POAF), characterized as AF that arises 1-3 days after surgery, occurs after 30%-40% of cardiac and 10%-20% of non-cardiac surgeries, and is thought to arise due to transient surgery-induced triggers acting on a preexisting vulnerable atrial substrate often associated with inflammation and autonomic nervous system dysfunction. Current experimental studies often rely on human atrial tissue samples, collected during surgery prior to arrhythmia development, or animal models such as sterile pericarditis and atriotomy, which have not been robustly characterized.

**Aim:**

To characterize the demographic, electrophysiologic, and inflammatory properties of a POAF mouse model.

**Methods and Results:**

A total of 131 wild-type C57BL/6J mice were included in this study. A total of 86 (65.6%) mice underwent cardiothoracic surgery (THOR), which consisted of bi-atrial pericardiectomy with 20 s of aortic cross-clamping; 45 (34.3%) mice underwent a sham procedure consisting of dissection down to but not into the thoracic cavity. Intracardiac pacing, performed 72 h after surgery, was used to assess AF inducibility. THOR mice showed greater AF inducibility (38.4%) compared to Sham mice (17.8%, *P* = 0.027). Stratifying the cohort by tertiles of age showed that the greatest risk of POAF after THOR compared to Sham occurred in the 12-19-week age group. Stratifying by sex showed that cardiothoracic (CT) surgery increased POAF risk in females but had no significant effect in males. Quantitative polymerase chain reaction of atrial samples revealed upregulation of transforming growth factor beta 1 *(TGF-β1)* and interleukin 6 (IL6) and 18 (IL18) expression in THOR compared to Sham mice.

**Conclusion:**

Here, we demonstrate that the increased POAF risk associated with CT surgery is most pronounced in female and 12-19-week-old mice, and that the expression of inflammatory cytokines is upregulated in the atria of THOR mice prone to inducible AF.

## One sentence summary

We developed a mouse model of POAF that replicates key features of this condition in humans in terms of incidence and inflammatory indices. We demonstrated that female mice have a greater POAF risk than males, highlighting the importance of considering biological sex in future POAF mouse studies.

## Graphical Abstract

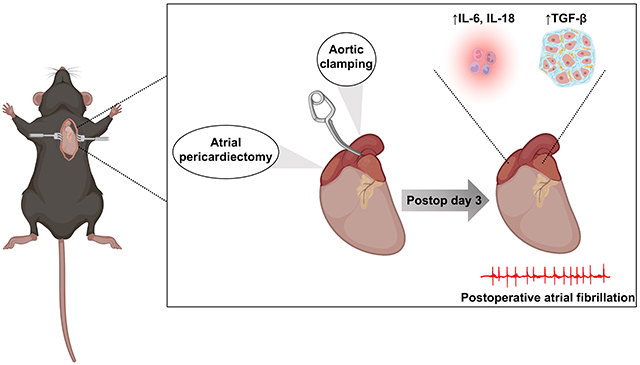

## INTRODUCTION

Atrial fibrillation (AF) is the most common arrhythmia and affects 4% of adults older than 60^[1]^. Common risk factors for AF include hypertension, valvular heart disease, heart failure, obstructive sleep apnea, obesity, and chronic kidney disease^[2]^. One important risk factor for AF is surgery, and AF developing during the days following surgery is termed postoperative AF (POAF). POAF typically occurs 1-3 days after surgery^[3]^ and portends a 5-fold higher risk of chronic AF^[4]^ and a 2-fold higher risk of stroke and mortality^[5,6]^. Rates of POAF are 15%-42% after cardiac surgery^[7]^ and 10%-20% after non-cardiac surgery^[6,8,9]^.

POAF is thought to arise due to transient triggers such as autonomic activation and post-surgical inflammation acting on a preexisting substrate to promote triggered activity and reentry^[9]^. Evidence for the role of autonomic activation comes from the significant reduction in POAF with beta-blocker therapy^[10]^ as well as higher circulating norepinephrine^[11]^ and aldosterone^[12]^ levels observed in POAF patients. Evidence for the role of inflammation is less consistent among experimental studies, but higher preoperative and postoperative white blood cell counts are seen in POAF patients^[13]^. Structural and cellular alterations such as left atrial fibrosis^[14]^ and decreased atrial autophagy^[15]^ have also been linked to POAF development in mice.

Experimental studies of POAF are limited. Human atrial tissue samples provide information about predisposing substrate but are typically collected during the surgical procedure days prior to POAF development and thus do not provide information about the substrate at the time of arrhythmia initiation. Current animal models of POAF include sterile pericarditis, pericardiotomy, and atriotomy^[16–18]^. However, unlike in the clinical setting with patients undergoing cardiac surgery, the currently available mouse models do not account for the ischemic and hemodynamic insults inherent to cardiac surgery. Moreover, to our knowledge, a comprehensive and powered analysis of the electrophysiologic (EP) and AF inducibility of these models in a large cohort of mice has not been conducted. Here, we describe a model of POAF consisting of a bi-atrial pericardiectomy with cross-clamping of the thoracic aorta to simulate cardiothoracic (CT) surgery. We systematically characterize the AF inducibility and inflammatory EP signature in our POAF model using over 100 wild-type mice.

## MATERIALS AND METHODS

### Mouse colonies:

All experiments were carried out using wild-type male and female C57BL/6J mice purchased from Jackson Labs (strain #000664).

### Experimental protocols:

Experiments were carried out according to NIH Guidelines on the Use of Laboratory Animals. All procedures were approved by the Baylor College of Medicine Institutional Animal Care and Use Committee. A total of 131 mice (48% male) were included in this study (45 Sham, 86 THOR). All animals were given one injection of buprenorphine subcutaneously at a dose of 1 mg/kg at least 1 h before surgery. Animals were monitored every day after surgery until terminal EP studies were performed 72 h after the surgery. Meloxicam, which is often administered every 24-48 h postoperatively in mice, was withheld per protocol due to the potential confounding effect of its non-steroidal anti-inflammatory mechanism of action on the pathways involved in POAF (see Discussion).

### Animal model:

**Figure 1.**
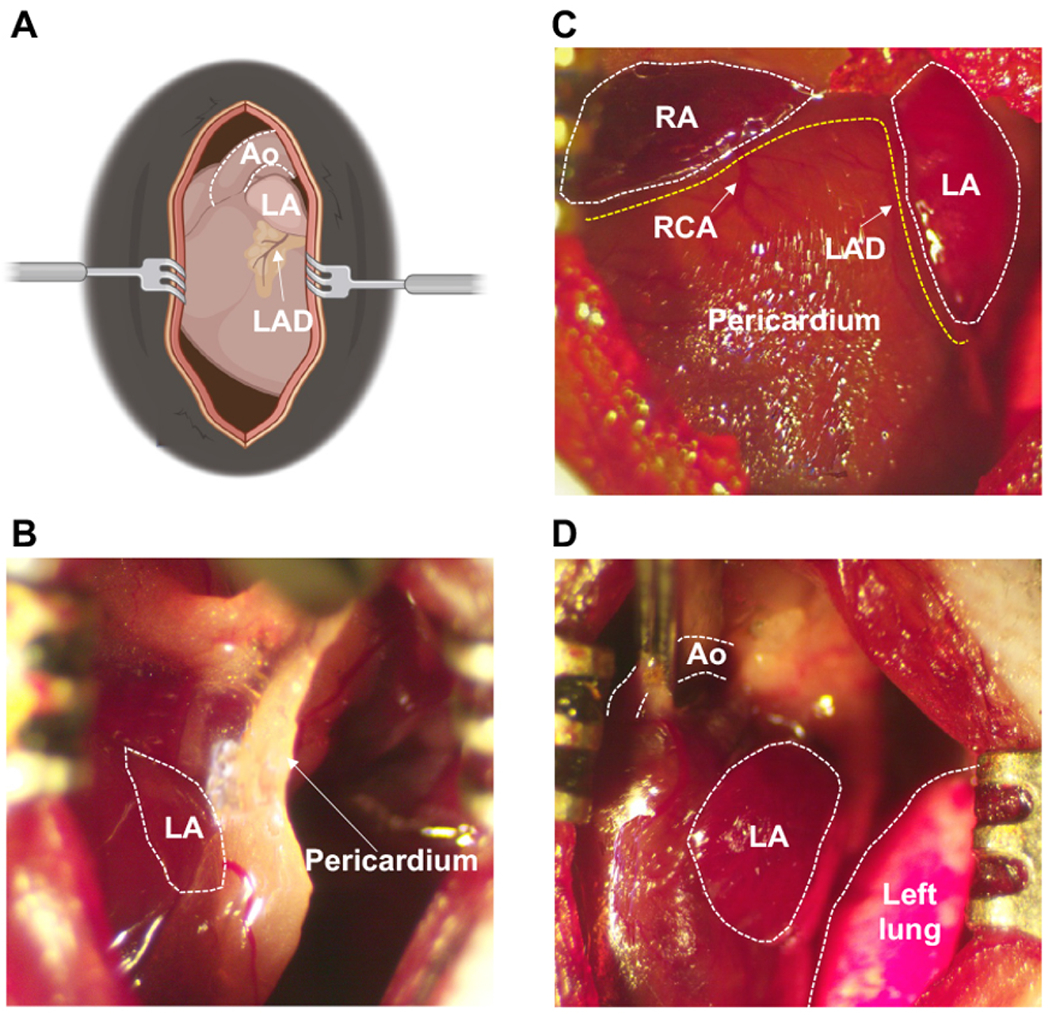
Cardiothoracic surgery workflow. (A) Schematic illustration of exposed chest cavity following dissection through 2nd or 3rd intercostal space. (B) Fibrous pericardium covering the left atrium prior to pericardiectomy. (C) Status after bi-atrial pericardiectomy for both atria, with pericardium removed. (D) Cross-clamping of ascending aorta. Ao: Aorta; LA: left atrium; LAD: left anterior descending coronary artery; RA: right atrium; RCA: right coronary artery.

Our mouse model of POAF was designed to recapitulate CT surgery [Figure 1]. Anesthesia was induced by placing mice in an induction chamber filled with 4% isoflurane in 100% oxygen until the mice lost consciousness. Mice were intubated and ventilated at a tidal volume of 150 μL and respiratory rate of 175 breaths/minute, and anesthesia was maintained using 2.5% v/v isoflurane in 100% oxygen. Body temperature was maintained at 37.0 °C ± 0.5 °C using a heated platform guided by a rectal thermometer. After dissection through the skin and subcutaneous tissue, the pectoralis major and minor muscles were separated laterally and the thoracic cavity was exposed through the 2nd or 3rd intercostal space [Figure 1A]. Bi-atrial pericardiectomy [Figure 1B and C] and cross-clamping of the thoracic aorta for 20 s [Figure 1D] were performed. For the sham procedure, the same skin and muscle dissection was performed, but the chest cavity was not entered.

### Programmed electrical stimulation:

72 h after CT surgery, AF inducibility was analyzed by performing a right heart catheterization to conduct intracardiac pacing as previously described^[19]^. Briefly, the mouse was anesthetized (1.75% v/v isoflurane in 100% oxygen). The subcutaneous tissue superficial to the right external jugular vein was dissected, and proximal and distal control of the right external jugular vein was secured with 6-0 sutures. A 1.1F octapolar catheter (EPR-800, Millar Instruments, Houston, TX) was then inserted into the right external jugular vein, through the superior vena cava, and into the right atrium and ventricle. Correct placement of the catheter was verified by looking for the appropriate *P* wave deflection after intra-atrial pacing and the appropriate QRS deflection after intra-ventricular pacing. All EP protocols were performed at 1.75% v/v, isoflurane/oxygen and rectal temperature between 37.0 °C ± 0.5 °C.

Following baseline recordings, the sinus node recovery time (SNRT) was determined by pacing the right atrium at a basic cycle length (BCL) of 100 milliseconds (ms) for 15 s and then measuring the amount of time from the last pacing impulse until the first spontaneous sinoatrial node beat^[20]^. The SNRT was repeated at a BCL of 90 ms. The atrial effective refractory period (AERP) was determined by applying a series of atrial pacing trains at a BCL of 100 ms, each with a coupled premature stimulus at the end (referred to as S2). The S1-S2 was decreased by 2 ms from 70 ms to 20 ms. The AERP was defined as the longest S1-S2 interval that failed to propagate into the ventricles. AF inducibility was determined by performing the A-burst protocol^[21]^, which applies a series of 2-s bursts starting at a BCL of 40 ms. Each 2-s burst decreases the BCL by 2 ms until a BCL of 20 ms is reached. The burst pacing protocol was performed in triplicate per mouse and AF inducibility was defined as a positive AF event lasting at least one second in at least two (out of three) positive events. A positive AF event was defined by irregularly irregular RR intervals without discernable P waves on electrocardiogram (ECG).

### RNA isolation and cDNA preparation:

Total RNA was isolated from the atrial appendages of six Sham mice without AF and six THOR mice with AF. Tissue was harvested immediately after EP pacing. Both the left and right atrial appendages were used for all samples. After harvesting, the heart underwent retrograde perfusion with cold phosphate-buffered saline. Atrial samples were then frozen in liquid nitrogen for 5 min and stored at −80 °C. To isolate total RNA, atrial samples were ground up and then 200 μL of TRIzol (Ambion) was added to each sample. Samples were spun down the samples for 60 s at 16,000 rpm. The Direct-zol RNA MiniPrep (Zymo Research) was used to isolate total RNA. RNA concentrations were measured using a nanodrop and stored at −80 °C. To generate cDNA, reverse transcription was carried out using the iScript Reverse Transcription Supermix (BioRad) with 500 ng of RNA per sample.

### qPCR:

Quantitative polymerase chain reaction (qPCR) was conducted for *IL-6, IL-1, IL-18, IL-10*, interferon gamma (*IFN-γ*), tumor necrosis factor alpha (*TNF-α*), and *TGF-β1* with the primers listed in Supplementary Table 1. Three technical replicates were performed per sample. Six individual biological replicates were used per group (six Sham and six THOR mice). A total of 10 μL reaction solution was used per well: 5 μL of SYBR Green Master Mix (Thermo Fisher Scientific), 0.5 μL of 4× Yellow Sample Buffer (Thermo Fisher Scientific), 1 μL of 10μM forward primer, 1 μL of 10 μM reverse primer, 2 μL of cDNA (prepped as described above), and 3 μL of distilled water. Fold changes in gene expression were calculated using the delta-delta Ct method as previously described^[22]^. Gene expression fold changes (relative to *GAPDH*) in THOR mice were then normalized to those in Sham mice by dividing by the average Sham fold change. To compare gene expression between Sham and THOR mice, two-sample *t*-tests were conducted comparing the delta-delta Ct values due to the non-linearity of the fold-change values.

### Statistical analyses:

All statistical analyses were performed using RStudio version 1.4.1717 and GraphPad Prism version 9.3.1. Two-sided *P* values less than 0.05 were considered statistically significant. Chi-square tests were performed for categorical independent and dependent variable data. Fisher’s exact tests were used in lieu of chi-square tests when expected counts were less than five in any group. Two-sample t-tests were performed for binary independent and continuous dependent variable data. Wilcoxon rank-sum tests were used in lieu of 2-sample t-tests when the dependent variable was ordinal (e.g., number of AF events). Outliers were defined as > 2 standard deviations above or below the mean.

## RESULTS

### Baseline characteristics:

A total of 131 mice (48% male) were included in the study - 45 Sham and 86 THOR. Intraoperative mortality was 4% (*N* = 5 deaths) due to bleeding complications. Mice who died intraoperatively were not included in final mouse counts or subsequent analyses. Mice ages ranged from 7- to 27-week-old (median age was 14 weeks). Mice were analyzed by sex and tertiles of age (≤ 11 weeks, 12-19 weeks, and ≥ 20 weeks). ECG metrics are shown by treatment group [Table 1], tertiles of age [Table 2], sex [Table 3], and AF inducibility [Table 4]. All pairwise comparisons between groups did not reach statistical significance except for the comparison of PR interval by age group (ANOVA *P* = 5.6E-05). Subsequent pairwise comparisons demonstrated that PR interval differed between the ≤ 11-week *vs*. ≥ 20-week (*P* = 5.2E-05) and the 12-19-week *vs*. ≥ 20-week (*P* = 0.016) age groups but not the ≤ 11-week *vs*. 12-19-week age group (*P* = 0.023) at Bonferroni significance (*P* < 0.017, results not shown).

### Overall AF inducibility:

**Figure 2.**
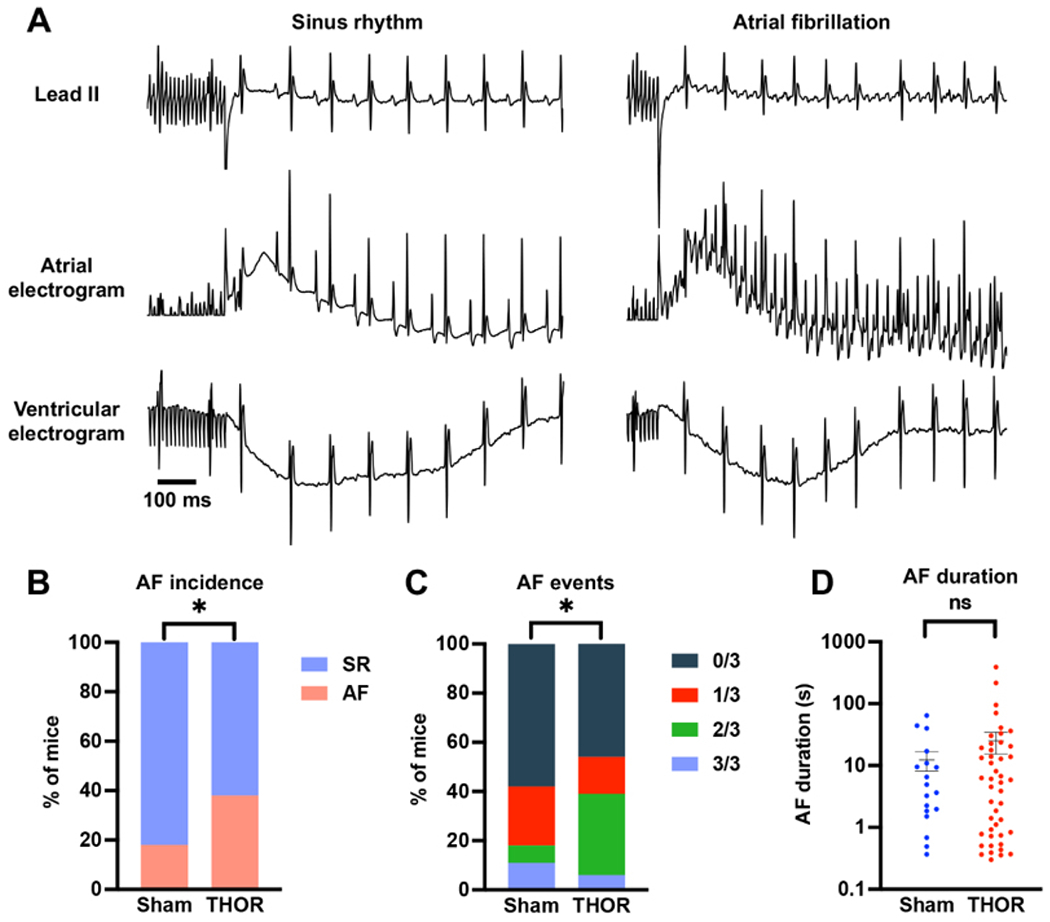
(A) Representative ECG tracings showing sinus rhythm (left) and AF (right) after the intracardiac burst atrial burst protocol. (B) Percent of mice in sinus rhythm (blue) and AF (red) in Sham (*n* = 45) and THOR (*n* = 86) mice. AF was defined as at least two (out of three) positive AF events after the A-burst protocol. (C) Percent of mice with zero, one, two, and three events after the A-burst protocol in Sham (*n* = 45) and THOR (*n* = 86) mice. (D) AF duration in Sham (*n* = 19) and THOR (*n* = 46) mice. Only mice with inducible AF episodes are included. Values plotted are equal to the sum of AF duration over the three A-burst protocols. Data are reported as mean ± SEM. *P* values in panels (B and C) were obtained from chi-square tests. *P* value in panel (D) was obtained from two-sample Student’s t-tests. *P < 0.05. *P* ≥ 0.05 denoted ns. ECG: Electrocardiogram; AF: atrial fibrillation; SR: sinus rhythm.

In the overall study, 33 out of 86 (38.4%) THOR mice exhibited inducible POAF, while 8 out of 45 (17.8%) Sham mice had POAF. Representative ECG tracings showing sinus rhythm and AF are shown in Figure 2A. The proportion of POAF events in THOR mice was greater than IN Sham mice (*P* = 0.027, Figure 2B). The number of inducible AF events (out of three) was higher in THOR *vs*. Sham mice (*P* = 0.0089, Figure 2C). These results were primarily driven by the greater number of THOR mice with 2 (out of 3) AF events [Figure 2C], since the proportion of THOR and Sham mice with 3 (out of 3) was similar. Of the mice who had POAF, the AF duration trended longer in THOR mice (mean = 34.9 s) compared to Sham (mean = 30.2 s), but the difference was not statistically significant (*P* = 0.41, Figure 2D).

### AF inducibility by age:

**Figure 3.**
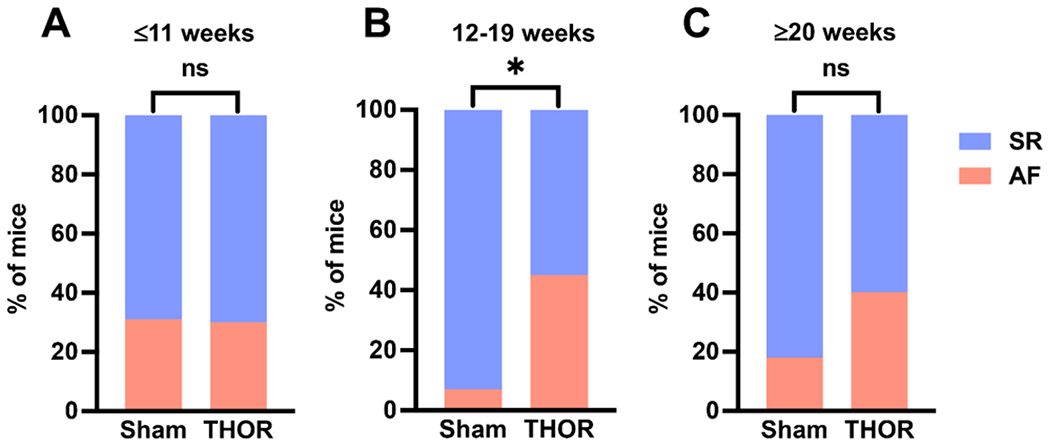
AF inducibility by age. (A) Percent of mice in sinus rhythm (blue) and AF (red) in Sham (*n* = 14) and THOR (*n* = 33) mice in the bottom age tertile. (B) Percent of mice in sinus rhythm (blue) and AF (red) in Sham (*n* = 14) and THOR (*n* = 34) mice in the middle age tertile. (C) Percent of mice in sinus rhythm (blue) and AF (red) in Sham (*n* = 17) and THOR (*n* = 20) mice in the oldest age tertile. AF was defined as at least two (out of three) positive AF events after the A-burst protocol. *P* values were obtained from chi-square tests. *P < 0.05. *P* ≥ 0.05 denoted ns. AF: Atrial fibrillation; SR: sinus rhythm.

Mice were analyzed according to tertiles of age to identify an age range at which the difference in AF inducibility after cardiac surgery differed the most between sham and THOR mice. Odds of inducible POAF in THOR were similar to Sham in the youngest tertile (odds ratio [OR] = 0.97) and greater than Sham in the middle (OR = 11.7) and older (OR = 3.11) tertiles [Figure 3A–C]. Only the middle tertile (12-19 weeks old) showed a statistically significant difference in AF inducibility (*P* = 8.2E-03, Figure 3B). The number of AF events was nominally significantly different between Sham and THOR mice in the youngest (*P* = 0.075) and middle (*P* = 0.053) tertiles [Supplementary Figure 1].

### AF inducibility by sex:

**Figure 4.**
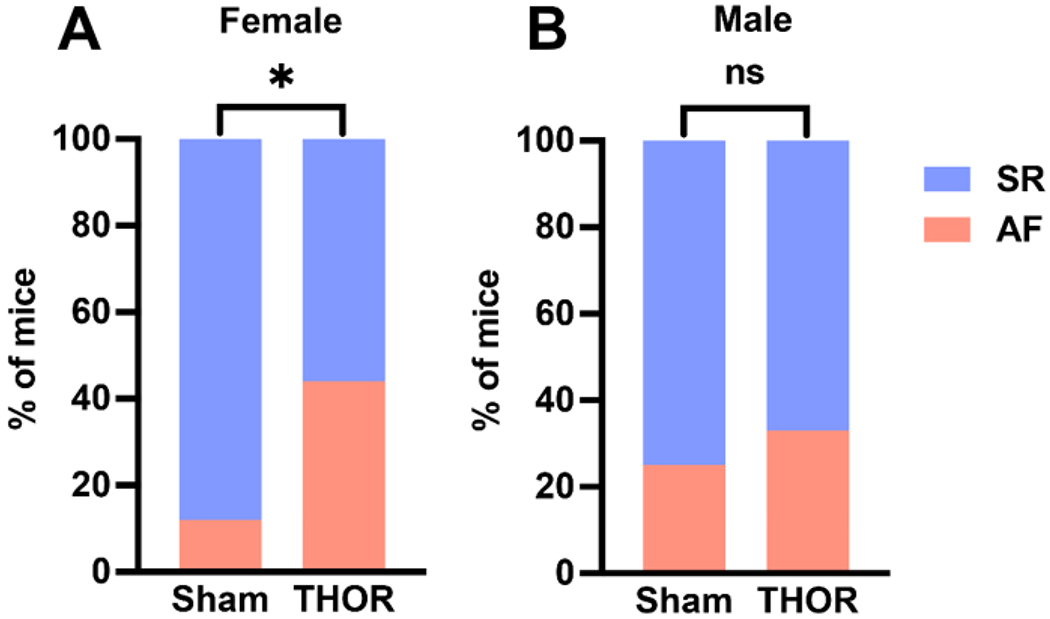
AF inducibility by sex. (A) Percent of female mice in sinus rhythm (blue) and AF (red) in *n* = 16 Sham and *n* = 52 THOR mice. (B) Percent of male mice in sinus rhythm (blue) and AF (red) in *n* = 10 Sham and *n* = 53 THOR mice. AF was defined as at least two (out of three) positive AF events after the A-burst protocol. *P* values were obtained from chi-square tests. *P < 0.05. *P* ≥ 0.05 denoted ns. AF: Atrial fibrillation; SR: sinus rhythm.

The odds of POAF were greater in male and female THOR mice. Only female mice exhibited statistically significantly greater AF inducibility after THOR compared to Sham (*P* = 6.9E-03, Figure 4A). There was no significant difference in AF inducibility between THOR and Sham male mice (*P* = 0.71, Figure 4B). Female THOR mice had a statistically significantly greater number of AF events compared to Sham (*P* = 0.039, Supplementary Figure 2A). There was no difference in the number of AF events for male Sham *vs*. THOR mice (*P* = 0.15, Supplementary Figure 2B).

### PCR amplification of inflammatory genes:

**Figure 5.**
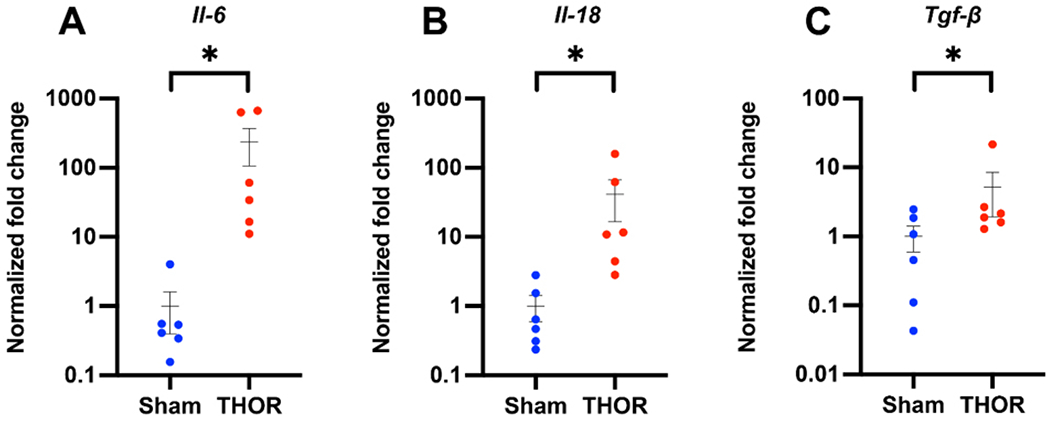
qPCR amplification of *IL-6, IL-18*, and *TGF-β1* mRNA levels in atrial tissue. Normalized fold changes of gene expression (relative to *Gapdh*) in *IL-6* (A), *IL-18* (B), and *TGF-β1* (C) calculated using the delta-delta CT method. *P < 0.05. *P* ≥ 0.05 denoted ns. *n* = 6 Sham, *n* = 6 THOR mice. qPCR: Quantitative polymerase chain reaction.

Gene expression of *IL-6*, *IL-1β*, *IL-18*, *IL-10*, *IFN-γ*, *TNF-α*, and *TGF-β1* was evaluated by qPCR in atrial tissue of six Sham and six THOR mice. GAPDH was used as the housekeeping gene. Normalized fold changes of gene expression (relative to the Sham group) are shown in Figure 5. Compared to Sham, THOR mice had a statistically significantly greater expression of *IL-6* (*P* = 1.82E-04, Figure 5A), *IL-18* (*P* = 1.7E-03, Figure 5B), and *TGF-β1* (*P* = 0.046, Figure 5C). There were no statistically significant differences in *IL-1β*, *IL-10*, *IFN-γ*, and *TNF-α* expression between Sham and THOR mice [Supplementary Figure 3].

## DISCUSSION

In this study, we characterized a novel mouse model of POAF in a cohort of 131 mice. The surgery-related mortality in our study was around five percent. All mortalities occurred intraoperatively due to bleeding complications at an incidence of ~4%, which is similar to that associated with coronary artery bypass grafting in humans (3%)^[23]^. No mice died during the 72 h postoperative period. The absolute risk of developing POAF during the first three days post-surgery was 38.4% (33/86) in THOR and 17.8% (8/45) in Sham mice, corresponding to a 2.87-fold increase in odds of POAF in THOR compared to Sham mice. This effect size is comparable to POAF in humans, which occurs in ~30% of patients after cardiac surgery^[24]^, although our study assessed the incidence of inducible but not spontaneous AF. The inducibility of AF is an index for the development of an AF-maintaining substrate and is commonly assessed in non-surgical mouse models since only a few (transgenic) mouse lines exhibit spontaneous AF^[25,26]^. As AF in mice requires supraphysiologic triggers, baseline POAF risk may be higher than those seen in humans, but this increase in risk may be negated by the healthy baseline status of mice in our study compared to humans undergoing cardiac surgery.

### Impact of age on POAF risk

We sought to explore putative differences in risk of POAF by age and sex. We first analyzed POAF incidence by tertiles of age. The risk of POAF was greatest in the middle (12-19-week) tertile (odds ratio [OR] = 11.7, *P* = 8.2E-03). While the overall incidence of POAF was greater in the oldest tertile, the difference in POAF incidence between Sham and THOR mice was smaller due to the higher POAF incidence in Sham mice. Older age is a well-established risk factor for AF in humans^[27]^, and POAF clinical risk score algorithms include age > 60 years old, which portends a 2-4-fold increase in POAF risk^[28]^. In mice, Jansen *et al.* showed that the incidence of inducible AF in mice positively correlates with age - from 20% in 20-week-old to 40% in 60-week-old WT mice^[29]^. Loss of natriuretic peptide signaling was implicated as a key driver of the increase in AF susceptibility in older mice due to increased atrial fibrosis and shortened action potential duration. Other studies have demonstrated similar increases in AF susceptibility and atrial fibrosis^[30]^, as well as attenuated heart rate variability (i.e., altered sympathetic/parasympathetic balance)^[31]^ with aging in mice.

### Impact of sex on POAF risk

In humans, the male sex is associated with a 3-fold greater risk of POAF after cardiac surgery^[32]^. However, biological sex was not included in the risk score published by Mariscalco *et al*. due to its poor prognostic value for arrhythmia development in the regression model^[28]^. Nonetheless, our study demonstrated that female mice had significantly greater odds of POAF (OR = 5.82) compared to males (OR = 1.45); this is seemingly the opposite of what one would expect from human observations. In humans, AF affects males (77.5 per 1000 person-years) more than females (59.5 per 1000 person-years)^[33]^. However, AF in females is associated with more severe clinical sequelae such as embolic stroke^[34]^ and mortality^[33]^. One explanation for this difference in clinical outcome is that females with AF are more likely affected by valvular heart disease while males with AF tend to be affected by coronary artery disease^[35]^. Valvular AF is well-known to portend a significantly greater risk of stroke (17-fold increase) compared to nonvalvular AF (5-fold increase)^[36]^. Since we used relatively young mice with no known propensity to valvular heart disease or coronary artery disease, the absence of these comorbidities may have shifted the sex-specific risk for POAF.

In our study, we assessed the risk of inducible AF, which determines the presence of an arrhythmia-permissive substrate rather than the occurrence of spontaneous triggers of arrhythmias. In humans, one might hypothesize that females have a more AF-prone substrate compared to males but perhaps lack the AF triggers. Indeed, the lower success of AF catheter ablation in females is thought to occur partly due to the greater atrial fibrosis seen in females with long-standing AF compared to females without AF. The same increase in fibrosis does not occur in males with long-standing AF^[37]^.

In our mouse model, *TGF-β*, a key pro-fibrotic regulator, was upregulated in THOR mice with POAF compared to Sham. While fibrosis may be thought of as a more indolent process not directly involved in POAF, our results may reflect the initial activation of myofibroblasts in response to the damage due to CT surgery. Indeed, prior studies have demonstrated that myofibroblasts are activated in mice within three days of cardiac insults such as ischemia^[38]^ and stretch^[39]^. To further explore the effects of sex, we conducted qPCR comparing POAF-free female *vs*. male mice [Supplementary Figure 4]. Results suggest that female sham mice have greater *TGF-β* expression than male shame mice (*P* = 0.039), perhaps suggesting greater baseline fibrosis in female mice. Taken together, our results showing greater POAF incidence in female mice may reflect a sex difference in the propensity toward atrial fibrotic remodeling following CT surgery.

### Role of inflammation in postoperative AF

Postoperative inflammation is an important risk factor associated with POAF^[40]^. To this end, we withheld meloxicam, which is usually given every 24-48 h postop, in our CT surgery model. We then sought to explore whether our model induces a local atrial inflammatory response; thus, we conducted a qPCR analysis to measure the expression of proinflammatory cytokines in atrial tissue. Results demonstrated statistically significant upregulations of *IL-6*, *IL-18*, and *TGF-β1* in THOR compared to Sham mice. We did not find increases in proinflammatory cytokines *IL-1β* and *TNF-α* in THOR mice. While partially attributable to the relatively large biological variance among samples, these results could also indicate that regulation of these cytokines occurs post-translationally and/or that systemic (rather than local) cytokine levels are more likely affected. Indeed, a recent study demonstrated lower IL-1β protein expression in atrial cardiomyocytes from POAF patients^[41]^ due to the pore-forming effects of gasdermin D, which increased systemic IL-1β levels. Nonetheless, prior studies have reported increases in inflammatory mediators such as TNF-α^[42]^, IL-6^[43]^, IL-1β^[44]^, and NLRP3^[45]^ activation in POAF. Exposure of human atrial cardiomyocytes to IL-1β has been shown to reproduce the POAF-associated cellular proarrhythmic phenotype characterized by delayed afterdepolarizations^[45]^. Altogether, we show evidence that our CT surgery model induces a local inflammatory response in the atria.

### Study limitations

Our study has several limitations. First, we quantified gene expression, which may not reflect protein expression and/or serum levels. Second, the relatively large within-group variance among biological replicates used in our qPCR experiments may have precluded the determination of additional statistically significant differences in gene expression. Third, we did not differentiate between left and right atrium. The expression of ion channels, inflammatory and fibrotic mediators, and thrombogenic proteins differs between atria in humans^[46]^ and mice^[47]^. Fourth, our study focuses on inducible, not spontaneous, AF, which is a surrogate parameter of the AF-maintaining substrate rather than a trigger. In addition, EP studies were conducted in anesthetized mice (at 1.75% v/v isoflurane/oxygen). While prior studies suggest that cardiovascular depression does not occur at an isoflurane dose of 1.75%^[48,49]^, it is possible that sub-physiologic alterations in cardiac conduction could have altered AF susceptibility. Fifth, the Sham mice in the youngest tertile had a relatively high incidence (44%) of AF. This was partly due to the male predominance (62%) in this age group, as three (out of the four) Sham mice with AF were male. Female Sham mice in this age group had an AF incidence of 16.7%, which is slightly lower than the overall AF incidence in Sham mice (17.8%). Finally, the mice used in our study had healthy atria at baseline, which precluded the assessment of the role of the preexisting atrial substrate which is created by risk factors and comorbidities in patients. Moreover, the mice in this study were relatively young (range 7-27 weeks) as the mean age of the oldest mouse tertile corresponded to a human age of ~31 years. Taken together, the healthy baseline state and relatively young mouse ages used in this study limit the clinical translatability of our results and may partially account for the different sex-specific POAF risk profiles in our mice compared to humans.

In conclusion, we characterized a mouse model of POAF induced by a CT surgical procedure. We show that the risk of POAF is most pronounced in females and 12-19-week-old mice. We also show that key fibrosis (*TGF-β1*) and inflammatory (*IL-6*, *IL-18*) genes are upregulated postoperatively in our model. Taken together, our POAF model recapitulates key aspects of human AF, and we provide evidence that future POAF mouse studies should consider the effects of sex and age.

## Supplementary Material

supplementary material

## Figures and Tables

**Table 1.** Baseline ECG and EP characteristics by treatment group

	Sham (mean ± SEM)	THOR (mean ± SEM)	*P* value
N (%)	45 (34.3%)	86 (65.5%)	NA
RR (ms)	117 ± 2.4	113 ± 1.4	0.078
P wave duration (ms)	12.0 ± 0.026	12.1 ± 0.015	0.56
PR (ms)	42.9 ± 0.57	41.8 ± 0.39	0.11
QRS (ms)	16.9 ± 0.37	17.2 ± 0.34	0.67
SNRT100 (ms)	139 ± 5.8	145 ± 5.1	0.47
AERP100 (ms)	48.6 ± 1.2	46.9 ± 1.0	0.25

ECG: Electrocardiogram; AERP: atrial effective refractory period; ms: milliseconds; SNRT: sinus node recovery time.

**Table 2.** Baseline ECG and EP characteristics by age

	≤ 11 weeks (mean ± SEM)	12-19 weeks (mean ± SEM)	≥ 20 weeks (mean ± SEM)	*P* value
N (%)	47 (35.9%)	47 (35.9%)	37 (28.2%)	NA
Female (%)	18 (38.3%)	29 (60.4%)	21 (56.8%)	NA
RR (ms)	112 ± 2.0	114 ± 1.8	118 ± 2.8	0.20
P wave duration (ms)	12.3 ± 0.027	12.0 ± 0.026	11.9 ± 0.034	0.47
PR (ms)	40.6 ± 0.49	42.2 ± 0.45	44.1 ± 0.64	5.6E-05
QRS (ms)	16.3 ± 0.39	17.3 ± 0.55	17.7 ± 0.30	0.091
SNRT100 (ms)	148 ± 6.6	150 ± 7.4	128 ± 5.0	0.059
AERP100 (ms)	48.2 ± 1.0	48.0 ± 1.3	46.0 ± 1.8	0.49

ECG: Electrocardiogram; AERP: atrial effective refractory period; ms: milliseconds; SNRT: sinus node recovery time.

**Table 3.** Baseline ECG and EP characteristics by sex

	Female (mean ± SEM)	Male (mean ± SEM)	*P* value
N (%)	68	63	NA
RR (ms)	115 ± 1.8	113 ± 1.7	0.45
P wave duration (ms)	12.0 ± 0.018	12.2 ± 0.021	0.36
PR (ms)	42.2 ± 0.44	42.1 ± 0.50	0.93
QRS (ms)	16.9 ± 0.30	17.3 ± 0.44	0.46
SNRT100 (ms)	145 ± 5.5	141 ± 5.6	0.63
AERP100 (ms)	47.9 ± 1.1	47.1 ± 1.1	0.64

ECG: Electrocardiogram; AERP: atrial effective refractory period; ms: milliseconds; SNRT: sinus node recovery time.

**Table 4.** Baseline ECG and EP characteristics by AF inducibility

	AF negative (mean ± SEM)	AF positive (mean ± SEM)	*P* value
N (%)	90 (68.7%)	41 (31.3%)	NA
Female (N [%])	46 (51%)	22 (53.6%)	NA
RR (ms)	114 ± 1.5	115 ± 2.2	0.80
P wave duration (ms)	12.0 ± 0.015	12.3 ± 0.026	0.22
PR (ms)	42.0 ± 0.40	42.4 ± 0.55	0.58
QRS (ms)	17.1 ± 0.35	17.0 ± 0.35	0.83
SNRT100 (ms)	140 ± 4.4	151 ± 7.9	0.27
AERP100 (ms)	47.4 ± 0.98	47.8 ± 1.3	0.81

ECG: Electrocardiogram; AF: atrial fibrillation; AERP: atrial effective refractory period; ms: milliseconds; SNRT: sinus node recovery time.

## Data Availability

All data will be available upon request to Wehrens XHT.
