# Supplementary material for: In-depth characterization of a mouse model of postoperative atrial fibrillation
Source: J Cardiovasc Aging. Author manuscript; Available in PMC 2022 Nov 3. (PMC9632544; doi:10.20517/jca.2022.21)
Supplement: supplementary material [file NIHMS1825260-supplement-supplementary_material.docx]

**Supplemental Material**

**In-depth characterization of a mouse model of postoperative atrial fibrillation**

Joshua A. Keefe, Jose Alberto Navarro Garcia, Li Ni, Svetlana Reilly, Dobromir Dobrev, Xander H.T. Wehrens

**Table S1**. Primers used for qPCR experiments.

| Gene | Forward primer (5’🡪3’) | Reverse primer (5’🡪3’) |
| --- | --- | --- |
| IL-6 | TGTGCAATGGCAATTCTGAT | GGTACTCCAGAAGACCAGAGGA |
| IL-1 | TGCAGTGGTTCGAGGCCTAAT | GTGACCACTCTCCAGTACCCAC |
| IL-18 | GTGAACCCCAGACCAGACTG | CCTGGAACACGTTTCTGAAAGA |
| IL-10 | AGCCTTATCGGAAATGATCCAGT | GGCCTTGTAGACACCTTGGT |
| IFN-γ | TCTTCAGCAACAGCAAGGCG | GCGACTCCTTTTCCGCTTCC |
| TNF-α | CAGGCGGTGCCTATGTCTCA | GGCTACAGGCTTGTCACTCG |
| TGF-β | TGTTAAAACTGGCATCTGA | GTCTCTTAGGAAGTAGGT |


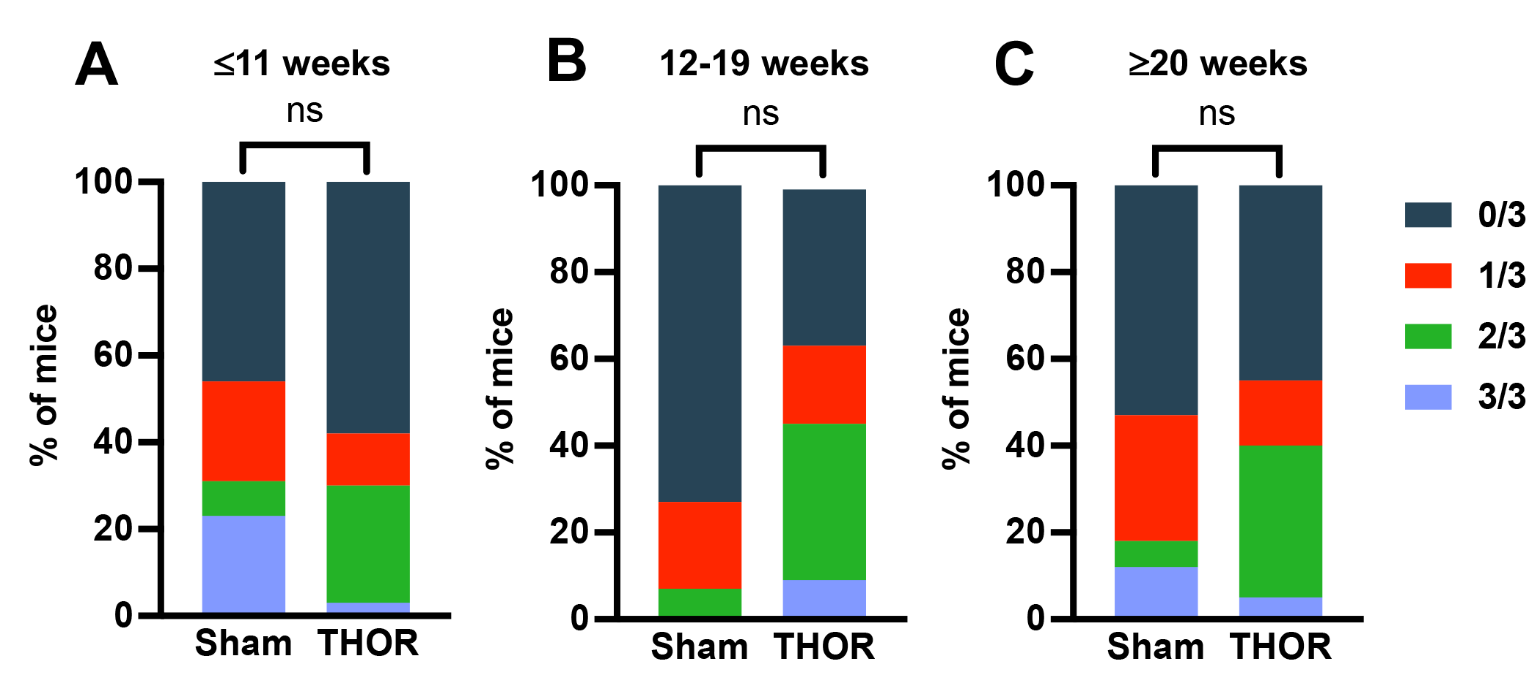


**Supplementary Figure 1**. Number of AF events (out of three) by age in the youngest (A), (B) middle, and (C) oldest tertiles. A positive AF event was defined as the presence of irregular R-R intervals without discernable P waves after the A-burst protocol (see methods). **P* < 0.05. *P* ≥ 0.05 denoted as “ns”


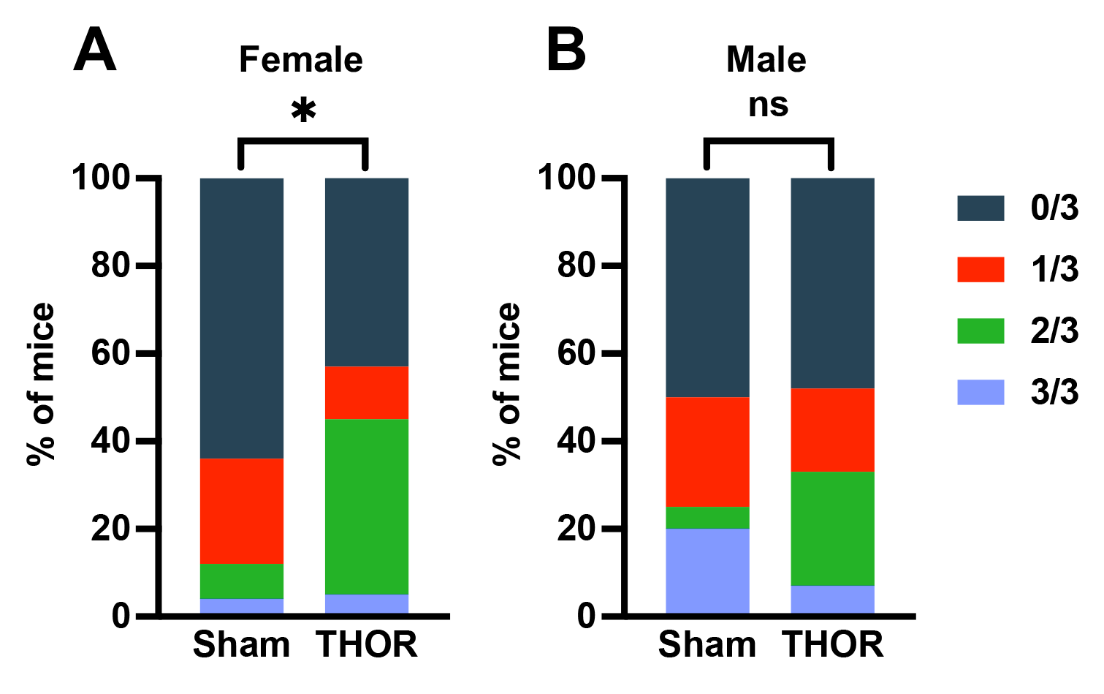


**Supplementary Figure 2**. Number of AF events (out of three) female (**A**) and male (**B**) mice. A positive AF event was defined as the presence of irregular R-R intervals without discernable P waves after the A-burst protocol. **P* < 0.05. *P* ≥ 0.05 denoted as “ns”


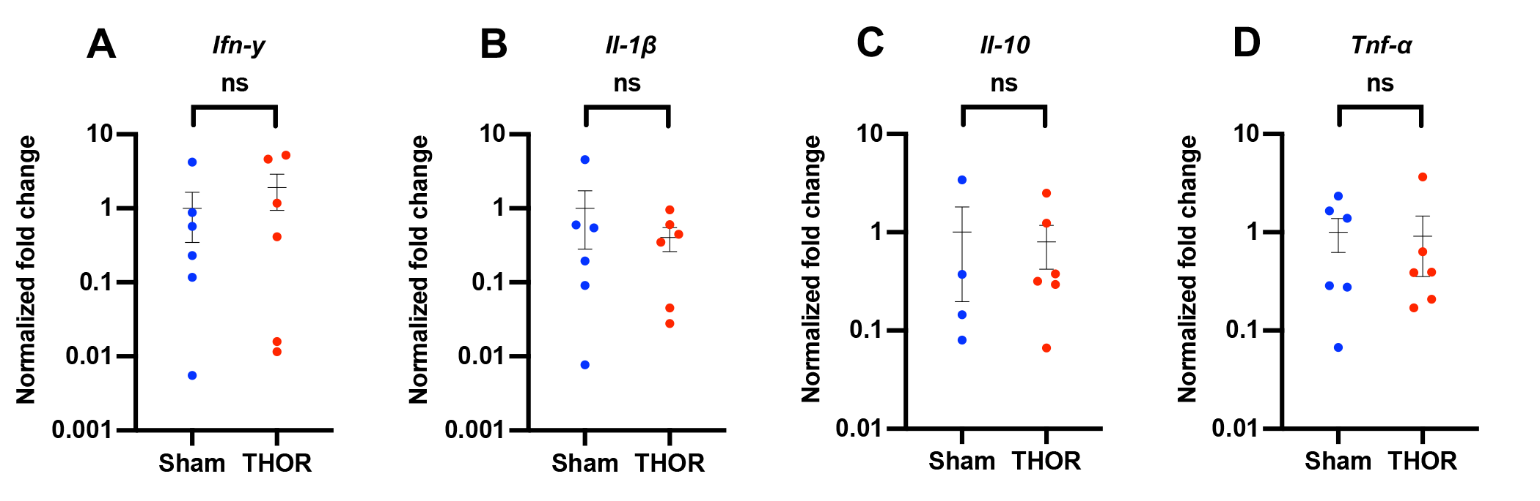


**Supplementary Figure 3**. qPCR amplification of *IFN-γ,* *IL-1β*, *IL-10*, and *TNF-α* mRNA expression levels in atrial tissue. Normalized fold changes of gene expression (relative to *GAPDH*) in *IFN-γ* (**A**)*,* *IL-1β* (**B**), *IL-10* (**C**), and *TNF-α* (**D**) calculated using the delta-delta Ct method. *P* ≥ 0.05 denoted as “ns”


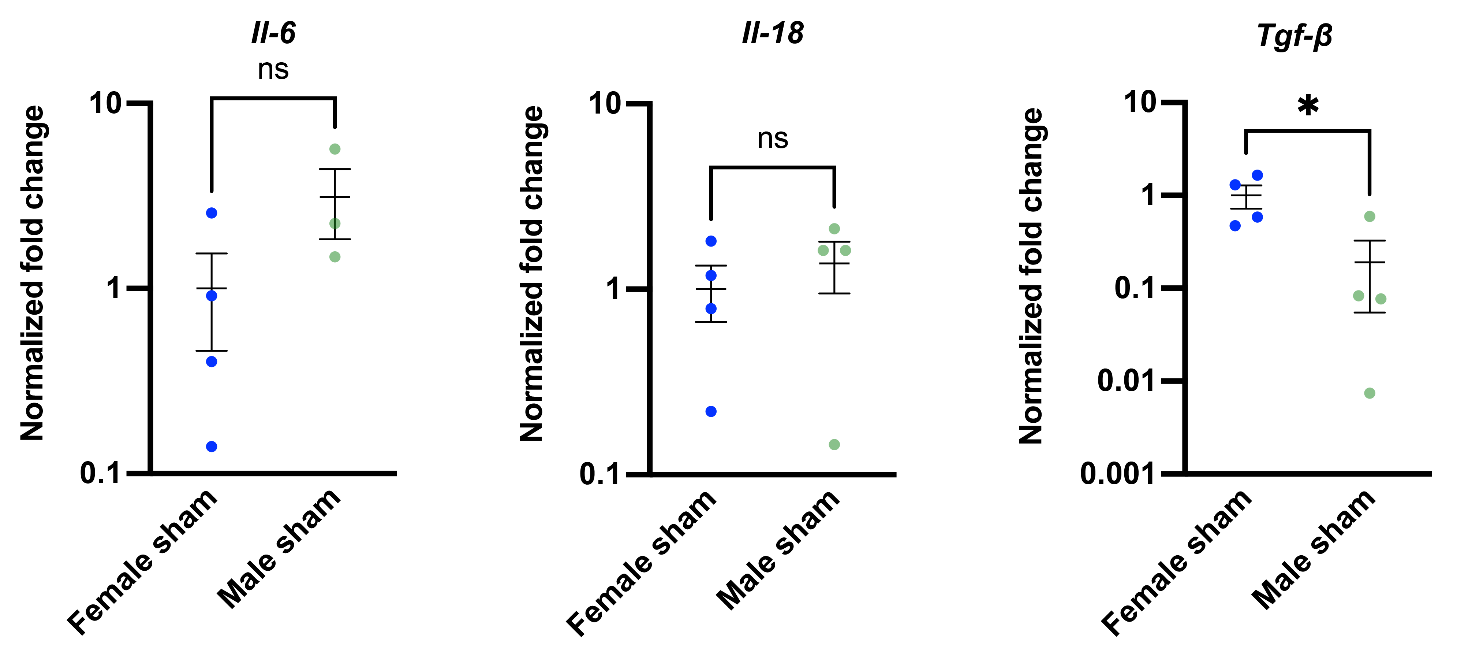


**Supplementary Figure 4**. qPCR amplification of *IL-6*, *IL-18*, and *TGF-β1* in POAF-negative female and male sham mice. Normalized fold changes of gene expression (relative to *GAPDH*) in *IL-6* (**A**), *IL-18* (**B**), and *TGF-β1* (**C**) calculated using the delta-delta Ct method. *P* ≥ 0.05 denoted as “ns”
